# Supplementary material for: Public perceptions of conflicting information surrounding COVID-19: Results from a nationally representative survey of U.S. adults
Source: PLoS One. 2020 Oct 21;15(10):e0240776. doi: 10.1371/journal.pone.0240776 (PMC7577476; doi:10.1371/journal.pone.0240776)
Supplement: S1 Table — (DOCX) [file pone.0240776.s001.docx]

**S1 Table. Issue-specific perceptions of disagreement among health experts and politicians about aspects of COVID-19 (coronavirus) (*N* = 1,007)**

|  | **Among health experts** | |  | **Among politicians** | |
| --- | --- | --- | --- | --- | --- |
|  | **Weighted %^a^** | **95% CI** |  | **Weighted %^a^** | **95% CI** |
| Who is most at risk of being infected with COVID-19 (coronavirus) | 28.2 | 24.6, 31.9 |  | 40.1 | 36.3, 44.0 |
| How dangerous it is for someone to become infected with COVID-19 (coronavirus) | 30.5 | 26.7, 34.2 |  | 49.8 | 45.8, 53.8 |
| Whether there is adequate access to testing for COVID-19 (coronavirus) | 53.5 | 49.5, 57.5 |  | 68.1 | 64.3, 71.9 |
| Whether the drugs chloroquine and hydroxychloroquine are effective in treating COVID-19 (coronavirus) | 57.5 | 53.5, 61.4 |  | 71.9 | 68.2, 75.7 |
| ^a^ Percentages are those who reported perceiving “some” or “a lot” of disagreement. | | | | | |
