# Supplementary material for: Public perceptions of conflicting information surrounding COVID-19: Results from a nationally representative survey of U.S. adults
Source: PLoS One. 2020 Oct 21;15(10):e0240776. doi: 10.1371/journal.pone.0240776 (PMC7577476; doi:10.1371/journal.pone.0240776)
Supplement: S2 Table — (DOCX) [file pone.0240776.s002.docx]

**S2 Table. Issue-specific perceptions of disagreement among health experts and politicians about the effectiveness of strategies for preventing the spread of COVID-19 (coronavirus) (*N* = 1,007)**

|  | **Among health experts** | |  | **Among politicians** | |
| --- | --- | --- | --- | --- | --- |
|  | **Weighted %^a^** | **95% CI** |  | **Weighted %^a^** | **95% CI** |
| Keeping 6 feet away from other people, except those you live with | 14.4 | 11.3, 17.4 |  | 23.8 | 20.4, 27.3 |
| Wearing a mask or other face covering when out in public | 23.3 | 19.8, 26.8 |  | 37.8 | 33.9, 41.7 |
| Keeping schools closed | 20.9 | 17.3, 24.4 |  | 38.2 | 34.3, 42.1 |
| Keeping all businesses closed except those considered essential (e.g., grocery stores, pharmacies) | 37.1 | 33.1, 41.0 |  | 65.9 | 62.1, 69.7 |
| Self-quarantining when sick | 9.7 | 6.9, 12.4 |  | 16.3 | 13.0, 19.6 |
| Washing your hands with soap several times per day | 5.9 | 3.5, 8.4 |  | 8.9 | 6.1, 11.7 |
| ^a^ Percentages are those who reported perceiving “some” or “a lot” of disagreement. | | | | | |
