# Supplementary material for: Public perceptions of conflicting information surrounding COVID-19: Results from a nationally representative survey of U.S. adults
Source: PLoS One. 2020 Oct 21;15(10):e0240776. doi: 10.1371/journal.pone.0240776 (PMC7577476; doi:10.1371/journal.pone.0240776)
Supplement: Attachment — Submitted filename: COVID conflict_PLOS ONE response to reviewers R2.docx [file pone.0240776.s004.docx]

# RESPONSE TO REVIEWERS

MANUSCRIPT:	Public perceptions of conflicting information surrounding COVID-19: Results

from a nationally representative survey of U.S. adults (PONE-D-20-22073R1)

DATE:	September 29, 2020

Thank you very much for the review of our manuscript, “Public perceptions of conflicting information surrounding COVID-19: Results from a nationally representative survey of U.S. adults.” Based on the reviewers’ helpful suggestions, we have made two additional improvements to the manuscript. Below we outline the concerns raised and our responses. In the revised manuscript, we indicate all revisions using track changes.

**Reviewer #1**

**The authors did an excellent job responding to reviewer comments. I look forward to seeing this published.**

Thank you very much; we appreciate your taking the time to review this work.

**Reviewer #3**

1) **The authors were very responsive to the reviews and have improved the manuscript. I just have two minor additional comments related to issues that were addressed in the response to the reviewers but that I believe could be addressed more directly in the manuscript.

   First, the authors state that some of the data not reported here are reported in a separate manuscript (Reviewer #3, Point 4). For transparency, I think the authors should cite the other paper (even if still in preparation or under review) and briefly describe the content so that reviewers/readers can be confident that the papers do not overlap.**

We now cite this paper, which was recently accepted for publication, and the following text has been added to p. 8: “Data that describe levels of public awareness of disparities in COVID-19 mortality and correlates of that awareness are reported elsewhere.[36]”

2) **Second, the authors describe the conceptual distinction between aspects and strategies related to COVID-19 in the letter (Reviewer #3, Point 6), but I did not see this described in the manuscript. I would recommend adding the justification from the letter to the manuscript, and perhaps including the correlations between the scales as well, in order to justify this decision to the reader.**

We thank the reviewer for this suggestion. The following text has been added to pp. 10-11: “For both the health expert and politician question blocks, we kept the aspects and strategies indices separate because of the conceptual distinctions between them. All six items in the strategies index assess the effectiveness of strategies for preventing the spread of COVID-19; the four items in the aspects index, though more loosely connected to one another, are concerned with perceptions of risk, testing, and treatment. The indices are not so strongly correlated so as to suggest they are capturing the same phenomena (aspects and strategies indices, health experts: r = 0.55; politicians: r = 0.55).”

Thank you again for the opportunity to revise and improve this manuscript. We are hopeful that this paper is now acceptable for publication in *PLOS ONE*.
